# On Dr. Adams's Claim to Priority in Suggesting Depletion in Hydrophobia, &c.

**Published:** 1814-04

**Authors:** 

**Affiliations:** Halesworth


*For the Medical and Physical Journal.*

### *On Dr. Adams's Claim to Priority in suggesting Depletion in Hydrophobia, &c.; by* Mr. Abel.

DR. ADAMS having noticed in the last Number of your Journal, my reply to his observations on Hydrophobia, in a very brief and rather supercilious manner, and having endeavored to produce an impression on the minds of his readers, that I had merely urged appearances as imputable to inflammation, which he had previously admitted; I beg leave, in proof of the propriety of my observations, to state what appeared to me the leading objects of his former communication.

The first object of Dr. Adams was to bring forward a claim to "the merit of priority" in suggesting the propriety of copious depletion in hydrophobia; his second, to prove that the appearances attributed by Mr. Borrett and others to inflammation of the stomach, were explicable on the supposition of digestion of that organ; his last, to lay before the public some novel but conjectural views of hydrophobia.

To the second part of Dr. A.'s paper I principally directed my attention, as being in my opinion the most important, since it bore directly on facts, the accurate comprehension of which must materially modify our views of the nature and effects of hydrophobia, and of all diseases that disorder the structure or functions of the stomach.

The point at issue was simply this, did the appearances observed by Mr. Borrett depend on inflammation or on digestion? Dr. Adams inclined to the opinion that they depended on digestion; I endeavored to prove that they were the result of inflammation. If Dr. Adams admitted these appearances to be the result of inflammation, why embarrass the question by endeavoring to prove them to be the consequences of digestion?

Dr. Adams remarks in his last paper, "that all the opinions formerly offered by him are formed on the supposition that the common symptoms of hydrophobia arise from inflammation of the stomach." Be this as it may, I did not reply to the doctor's general opinions, but to his particular observations on Mr. Borrett's paper.

I quite agree with Dr. Adams, that every controversy like this must become tedious to your readers; and I certainly should not now trespass on their patience and your indulgence, was I not of opinion that Dr. Adams had placed the subject of discussion in an incorrect point of view.

Before I quit my pen, I beg to remark, that I cannot but consider it illegitimate to compare effects, great in proportion to the previous health of an organ,' to appearances discoverable in the organ after it has been unquestionably implicated in the ravages of a most destructive disease.

*Halesworth,* CLARKE ABEL.

*March* 5, 1814.

